# Levodopa Responsive Parkinsonism in Patients with Hemochromatosis: Case Presentation and Literature Review

**DOI:** 10.1155/2017/5146723

**Published:** 2017-03-23

**Authors:** Tarun Girotra, Abhimanyu Mahajan, Christos Sidiropoulos

**Affiliations:** ^1^Department of Neurology, Henry Ford Hospital, Detroit, MI, USA; ^2^Department of Neurology and Ophthalmology, Michigan State University, East Lansing, MI, USA

## Abstract

Hemochromatosis is an autosomal recessive disorder which leads to abnormal iron deposition in the parenchyma of multiple organs causing tissue damage. Accumulation of iron in the brain has been postulated to be associated with several neurodegenerative diseases including Parkinson's disease. The excess iron promotes Parkin and *α*-synuclein aggregation in the neurons. Excess iron has also been noted in substantia nigra on MRI especially using susceptibility weighted imaging in patients with Parkinson's disease. We present a case of a young male with alleles for both C282Y and H63D who presented with signs of Parkinsonism and demonstrated significant improvement with levodopa treatment.

## 1. Short Communication

Hemochromatosis is an autosomal recessive disorder [1] which leads to abnormal iron deposition in the parenchyma of multiple organs causing tissue damage. Though iron is essential for several cellular functions including electron transport chain, citric acid cycle, neurotransmitter synthesis, and myelogenesis [2], excess iron can cause damage to the tissue by causing protein peroxidation, lipid peroxidation, and DNA oxidation via free radical damage. This can eventually lead to cellular and neuronal damage or death [3, 4]. The gene responsible for hemochromatosis is the HFE within Human Leukocyte Antigen (HLA) class I region on chromosome 6. HFE is a ligand for TfR [5]. Interaction between TfR and HFE leads to decreased affinity with which the receptor binds transferrin and this interaction may also modulate iron entry into the cell. When a mutant or nonfunctional variant of the HFE gene is present, ferritin levels are not under influence of a normal and functional HFE gene, which leads to enhanced accumulation of iron in peripheral tissues. In the brain, HFE is expressed in the choroid epithelial cells, endothelial cells in the microvasculature, and ependymal cell lining within the brain where it can affect iron accumulation in the brain [6, 7]. Accumulation of iron in the brain has been postulated to be associated with several neurodegenerative diseases including Parkinson's disease [8]. The excess iron promotes Parkin and *α*-synuclein aggregation in the neurons [8–10]. Excess iron has also been noted in substantia nigra on MRI especially using susceptibility weighted imaging (SWI) in patients with Parkinson's disease [10]. Substantia nigra is more vulnerable to excessive iron accumulation than other surrounding regions. Increased iron in the substantia nigra is associated with ineffective handling of reactive oxygen species and, therefore, oxidative stress. Excess iron can further lead to direct injury to the mitochondria [11]. A brief literature review using the terms “hemochromatosis” and “Parkinson's disease” revealed that only 5 cases of concomitant Parkinson's disease and hemochromatosis have been reported before [12, 13]. The largest case series among them consisted of four cases with preexisting hemochromatosis, developing idiopathic Parkinson's disease between the ages of 55 and 63 years [12]. Three out of the four cases described in this series had C282Y homozygous mutations.

We present the case of a 41-year-old right handed Caucasian male who came to our clinic with a six-month history of bilateral hand rest and action tremor, more pronounced on the right. Further inquiry revealed associated symptoms including anosmia, shoulder stiffness, difficulty rising up from a seated position, depressed mood, and fatigue. His past medical history was notable for hemochromatosis, migraines, restless leg syndrome, and obstructive sleep apnea. He has compound heterozygosity for the genetic hemochromatosis gene in that he has alleles for both C282Y and H63D. Family history was significant for Parkinson's disease in his mother, which first manifested when she was around 50 years of age.

His neurological exam demonstrated normal higher mental function, flat affect, masked-facies, normal cranial nerves, moderate rigidity in right arm, mild rigidity in left arm, bilateral bradykinesia on repetitive finger tapping, and mixed resting and action tremors in the right and left hand. The remainder of motor, sensory, and cerebellar exam was unremarkable. His gait was narrow-based with normal stride but diminished right arm swing. A levodopa challenge with sequential escalation of doses led to mild improvement in his appendicular symptoms.

**Figure 1:**
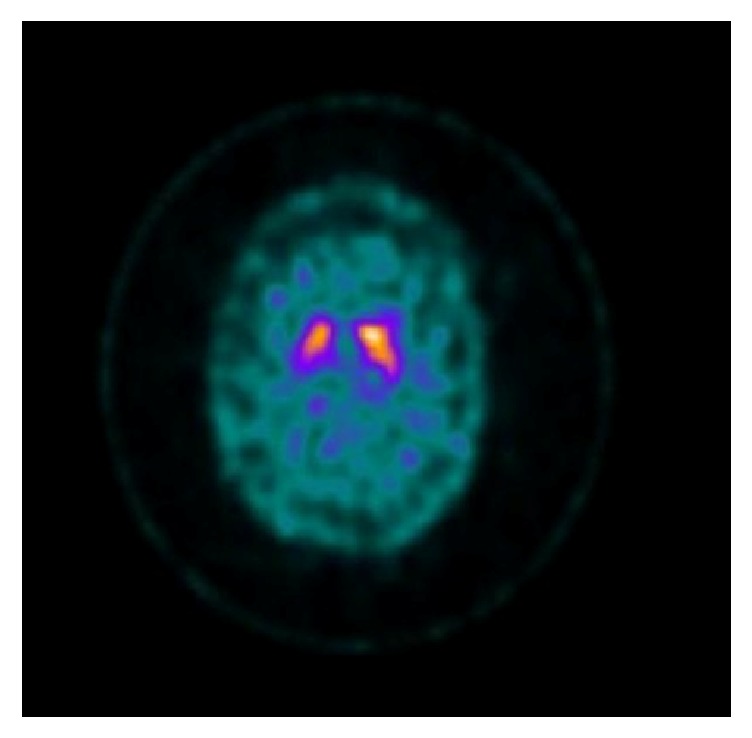
DaTscan shows asymmetric radiotracer uptake with mildly decreased uptake in the right putamen.

A brain MRI was done which was unremarkable whereas single-photon emission computed tomography (SPECT) brain imaging with I-123 ioflupane (“DaTscan”) revealed asymmetric radiotracer uptake with mildly decreased uptake in the right putamen [Figure 1].

Over the following eight months the patient showed modest but clear improvement in symptoms on treatment with oral carbidopa-levodopa 25–100 mg slowly escalated to three tablets four times a day.

Apart from our case, only one other case describes an onset of Parkinsonian symptomatology at a young age [13], thereby contributing to the literature on this controversial topic.

Given the theoretical risk of increased Parkinsonism in patients with disorders of iron regulation, several population based studies have been done to determine the association between hemochromatosis and its genetic mutations with Parkinson's disease but the results have been conflicting. One Norwegian and two Italian studies failed to find any significant association between the frequencies of C282Y, H63D, and S65C mutations in Parkinson's disease group and control group [14–16]. A Dutch study observed increased risk of Parkinson's disease in patients with C282Y gene mutation [17]. A Portuguese population based study also noted increased frequency of C282Y carrier status in Parkinson's disease cohort [18]. An Australian study however observed that C282Y allele could be protective against Parkinson's disease [19], contrary to the priorly mentioned Dutch and the Portuguese studies. Among these studies, only one study by Biasiotto et al. [16] looked at the age of onset between Parkinson's disease patients with no HFE mutations or at least one mutation associated with hemochromatosis. They did not find any significant difference between the age of onset among other factors such as clinical presentation and response to levodopa. They concluded that, in the Italian population, the most common HFE mutations have no specific influence on the clinical features of the disease and are not associated with a higher risk of developing the disease.

While there exists a plausible risk of neurodegenerative disease related to aberrant iron metabolism, several population based studies have failed to ascertain a more definite association between HFE gene mutations in Parkinson's disease populations.

The interplay between certain environmental and genetic factors may predispose certain patients suffering from hemochromatosis to develop Parkinson's disease at a younger age. Based on the data discussed above, future directions should include analyzing HFE mutation frequencies in patients with early onset Parkinsonism to gather more evidence towards this hypothesis.
